# Knockdown of RPL28 Inhibits the Progression of Clear Cell Renal Cell Carcinoma

**DOI:** 10.1002/cnr2.70683

**Published:** 2026-09-08

**Authors:** Runbiao Luo, Guozhong Wu, Zhouzhou Xie, Yuchen Xiao, Wenbiao Zhu, Huiming Jiang

**Affiliations:** ^1^ Department of Urology, Meizhou People's Hospital Meizhou Academy of Medical Sciences Meizhou People's Republic of China; ^2^ Affiliated Meizhou Hospital of Shantou University Medical College Meizhou People's Republic of China; ^3^ Shantou University Medical College Shantou People's Republic of China; ^4^ Department of Pathology Affiliated Meizhou Hospital of Shantou University Medical College Meizhou People's Republic of China

**Keywords:** biomarker, clear cell renal cell carcinoma, prognosis, RPL28

## Abstract

**Background:**

Clear cell renal cell carcinoma (ccRCC) is the most common and aggressive histological subtype of kidney cancer. Previous studies have implicated ribosomal protein L28 (RPL28) in the progression of various cancers; however, its role in ccRCC remains unclear.

**Aims:**

This study aimed to investigate the expression, prognostic significance, and potential biological role of RPL28 in ccRCC.

**Methods and Results:**

RPL28 expression data and corresponding clinicopathological information were obtained from the UCSC Xena platform, and pan‐cancer expression patterns were assessed using TIMER2.0. RPL28 expression was further validated in clinical ccRCC tissue samples. Prognostic, immune‐infiltration, and drug‐sensitivity analyses were performed, and the biological effects of RPL28 were evaluated using lentivirus‐mediated knockdown in ccRCC cells. RPL28 was significantly upregulated in ccRCC tissues and was associated with unfavorable overall survival (OS) and disease‐specific survival (DSS), as well as adverse clinicopathological features, including higher histological grade, advanced *T* stage, distant metastasis, and advanced TNM stage. Immune analyses revealed associations between RPL28 expression and selected features of the ccRCC immune microenvironment, while pharmacogenomic analyses suggested potential associations between RPL28 expression and predicted sensitivity to selected targeted agents. In vitro, RPL28 knockdown significantly inhibited ccRCC cell proliferation and migration. Gene set enrichment analysis (GSEA), together with experimental findings, further suggested that RPL28 may be involved in epithelial–mesenchymal transition (EMT)‐related processes in ccRCC.

**Conclusion:**

RPL28 is overexpressed in ccRCC and is associated with poor survival outcomes, malignant cellular phenotypes, EMT‐related signaling, selected immune‐microenvironmental features, and predicted therapeutic‐response phenotypes. These findings suggest that RPL28 may serve as a prognostic biomarker and potential therapeutic target in ccRCC, although further mechanistic and clinical validation is required.

## 1 Introduction

Renal cell carcinoma (RCC) constitutes a major malignancy within the urinary system, representing roughly 3.8% of cancer diagnoses in adults. Clear cell renal cell carcinoma (ccRCC) stands as the predominant subtype, comprising approximately 75% of RCC cases [1]. Based on the 2023 Cancer Statistics Report, the United States is projected to see 81 800 new kidney cancer cases, with 14 890 related deaths [2]. Traditional treatments such as radiotherapy and chemotherapy demonstrate limited effectiveness against ccRCC, with surgery remaining the primary therapeutic option. Despite advancements in molecular‐targeted therapies and immunotherapy, many patients still face poor prognoses. Over 50% of patients develop metastasis following nephrectomy, with the 5‐year survival rate for metastatic RCC staying under 12% [3]. This highlights the critical necessity for innovative prognostic biomarkers and therapeutic targets to enhance patient outcomes.

Ribosomes serve an essential function in cellular protein synthesis. Within eukaryotic organisms, ribosomes are composed of two distinct subunits: the large 60S subunit, containing 5S rRNA, 28S rRNA, 5.8S rRNA, and roughly 46 ribosomal proteins, and the small 40S subunit, harboring 18S rRNA and approximately 33 ribosomal proteins [4]. In addition to their traditional role in protein translation, ribosomal proteins are involved in extraribosomal functions such as cellular proliferation, apoptosis regulation, DNA repair, and cell cycle control [5]. Recent studies have indicated that ribosomal proteins contribute to tumorigenesis and the progression of various cancers [6].

Ribosomal protein L28 (RPL28), located on chromosome 19q13.42, belongs to the ribosomal protein family and has been implicated in several cancers. RPL28 exhibits elevated expression levels in colorectal cancer, demonstrating correlation with microsatellite instability, and its mutations are linked to poor outcomes in metastatic colorectal cancer [7, 8, 9]. Additionally, studies by Shi et al. have demonstrated that RPL28 contributes to resistance to the targeted agent sorafenib in liver cancer by inhibiting apoptosis [10]. However, the biological meaning of RPL28 in ccRCC cannot be directly inferred from these tumor types because ccRCC has a distinct molecular background characterized by VHL‐driven pseudohypoxia, metabolic reprogramming, frequent chromosome 3p alterations involving PBRM1 and BAP1, abundant immune infiltration, and pronounced intratumoral heterogeneity [11, 12, 13, 14]. These characteristics create a unique cellular context in which ribosomal components may affect cancer progression not only by supporting global protein synthesis, but also through selective translation, ribosome biogenesis, and ribosome heterogeneity. Ribosome heterogeneity refers to context‐dependent variation in ribosomal composition, ribosomal‐protein usage, rRNA modification, and associated translational programs, which may enable tumor cells to preferentially translate mRNAs involved in proliferation, stress adaptation, epithelial‐mesenchymal transition (EMT), and therapeutic resistance [15, 16]. Recent ccRCC studies using single‐cell, spatial‐transcriptomic, and multi‐omics approaches have further highlighted the importance of tumor‐cell plasticity, metabolic remodeling, and immune‐microenvironmental heterogeneity in disease progression [17, 18]. Nevertheless, whether the individual ribosomal component RPL28 is aberrantly expressed in ccRCC, whether it has prognostic significance beyond established clinicopathological factors, and whether it functionally participates in ccRCC‐cell proliferation and migration remain unclear. Therefore, the present study sought to examine the link between RPL28 expression and clinicopathological features, along with its potential utility as a prognostic indicator in ccRCC. Additionally, this investigation examined data from multiple databases and conducted loss‐of‐function experiments in vitro using ccRCC cell lines. Our findings suggest that RPL28 may act as a tumor promoter and could potentially function as a prognostic marker for unfavorable survival outcomes in patients with ccRCC.

## 2 Materials and Methods

### 2.1 Data Collection and Processing

RNA sequencing information and associated clinical‐pathological data for ccRCC were procured from the UCSC Xena platform (https://xena.ucsc.edu/) [19]. RPL28 expression values were analyzed as log2(FPKM +1)‐transformed RNA‐seq values. This dataset included 535 tumor specimens and 72 adjacent normal kidney tissues. Because all available tumor specimens were compared with all available adjacent normal tissues, the TCGA comparison was treated as an unpaired group‐level comparison. After the alignment of gene expression profiles with clinical information and the removal of cases having follow‐up periods shorter than 1 month, 518 participants remained for survival evaluation. Patients were divided into high‐ and low‐RPL28 expression groups according to the median expression value. Comparative examination of clinical and pathological characteristics between these groups was conducted. Survival outcomes were evaluated through Kaplan–Meier analysis and Cox regression modeling to determine the link between RPL28 expression and patient prognosis in ccRCC. To further validate RPL28 expression differences, we analyzed the International Cancer Genome Consortium (ICGC) RECA‐EU ccRCC cohort, including 91 tumor samples and 45 normal kidney samples [20]. The Gene_DE module of TIMER2.0 was employed to examine RPL28 expression across various cancer types [21].

### 2.2 Tissue Specimens

For clinical validation, paired ccRCC and adjacent non‐malignant kidney tissues were obtained from 12 patients who underwent partial or total nephrectomy at Meizhou People's Hospital. The mean age of the participants was 62.3 years. Histopathologically, eight cases (66.7%) were categorized as well‐differentiated (G1/2), and four cases (33.3%) were poorly differentiated (G3/4). Nine patients (75%) exhibited localized Stage I/II tumors, whereas three patients (25%) had advanced Stage III/IV disease. All participants furnished written informed consent in compliance with the Declaration of Helsinki, and the research was sanctioned by the Medical Ethics Committee of Meizhou People's Hospital (Approval no. 2023‐C‐26).

### 2.3 Mutation‐Stratified Analysis of RPL28 Expression

To further evaluate the association between RPL28 expression and major molecular alterations in ccRCC, somatic mutation data from the TCGA‐KIRC cohort were integrated with transcriptomic data. Patients were stratified into mutant and wild‐type groups according to the mutation status of representative ccRCC driver genes, including VHL, PBRM1, SETD2, BAP1, KDM5C, MTOR, TP53, PTEN, PIK3CA, and ARID1A. RPL28 expression was compared between mutant and wild‐type tumors.

### 2.4 Immune Infiltration Analysis

Considering the immunotherapy‐sensitive nature of ccRCC, we further evaluated the association between RPL28 expression and tumor immune infiltration. Immune‐cell abundance in the TCGA‐KIRC cohort was estimated using xCell algorithms. To reduce the potential confounding effect of tumor purity on bulk RNA‐seq‐based immune analyses, tumor‐purity‐adjusted immune‐cell infiltration scores and immune‐checkpoint gene‐expression values were further analyzed. Tumor purity estimates were obtained from the ESTIMATE algorithm. Tumor‐purity adjustment was performed using linear regression residualization with tumor purity as a covariate. To further explore immunological relevance, the relationship between RPL28 expression and representative immune‐checkpoint‐related genes (including PDCD1, LAG3, TIGIT, CTLA4, CD274, and PDCD1LG2) was also compared between the two RPL28 expression groups.

### 2.5 Drug‐Sensitivity Prediction Analysis

To explore the potential relationship between RPL28 expression and therapeutic sensitivity, predicted drug response was estimated using the oncoPredict R package with pharmacogenomic training datasets derived from CTRP [22]. Differences in predicted drug‐response scores between RPL28‐high and RPL28‐low groups were assessed. Spearman correlation analysis was also used to examine continuous associations between RPL28 expression and predicted drug‐response scores.

### 2.6 Immunohistochemistry

Immunohistochemical (IHC) analysis was conducted on paraffin‐embedded tissue samples using an anti‐RPL28 primary antibody (1:500 dilution, Abcam). Stained sections were evaluated by two independent pathologists. Comprehensive IHC assessment was executed following the methodology outlined previously [23].

### 2.7 Quantitative Reverse Transcription Polymerase Chain Reaction (qRT‐PCR)

Total RNA was procured from ccRCC cells employing an RNA extraction kit (Haigene Biotech Co. Ltd., China), followed by reverse transcription to complementary DNA (cDNA) with the PrimeScript RT reagent kit (Takara Bio Inc., Dalian, China). Quantitative PCR (qPCR) was conducted utilizing the SYBR Green PCR kit (Takara Bio Inc.). RPL28 relative expression levels were determined through the 2^−ΔΔCt^ methodology, with β‐actin functioning as the internal control. The primer sequences employed were: RPL28 (forward: 5′‐CAGACCTACAGCACTGAGC‐3′; reverse: 5′‐CTTGTTGATGGTGGTCCGCA‐3′) and β‐actin (forward: 5′‐CGAGCACGGCATCGTCAC‐3′; reverse: 5′‐CTGGATAGCAACGTACATGGC‐3′).

### 2.8 Cell Culture and Cellular Experiments

The ccRCC cell lines 786‐O and OSRC‐2 were procured from the Chinese Academy of Sciences. These cells were maintained in RPMI‐1640 medium comprising 10% fetal bovine serum and 1% penicillin–streptomycin and kept at 37°C in a humidified atmosphere with 5% CO_2_. Short hairpin RNA (shRNA) targeting RPL28 (NM_000991.5) was synthesized with the sequence 5′‐CTACAGCACTGAGCCCAATAA‐3′. Lentiviral vectors encoding RPL28‐specific shRNA (shRPL28) or control shRNA (shNC) were introduced into ccRCC cells. After 48–72 h, stable clones underwent selection utilizing puromycin, and the efficiency of RPL28 knockdown was confirmed through qRT‐PCR. Cell proliferation was examined employing the Cell Counting Kit‐8 (CCK‐8) assay. For the wound‐healing assay, 786‐O and OSRC‐2 cells were seeded into six‐well plates and cultured until they reached near‐confluence. A linear wound was generated using a sterile 200‐μL pipette tip. Cells were then cultured in serum‐reduced medium to minimize the contribution of proliferation to wound closure. The underside of each well was pre‐marked to ensure that images at 0 and 24 h were captured from the same location. Wound images were obtained using an inverted microscope, and scale bars were added to the representative images. Quantification was performed using ImageJ by measuring the wound area at 0 and 24 h. The wound‐closure rate was calculated as [(area at 0 h—area at 24 h)/area at 0 h]. Assays were performed using three independent biological replicates.

### 2.9 Functional Enrichment Analysis

To investigate the molecular pathways linked to RPL28 expression in ccRCC, gene set enrichment analysis (GSEA) was executed. The single‐sample GSEA method was applied to evaluate the activity of various gene sets. Spearman correlation analysis was utilized to examine the connection between RPL28 levels and the enriched pathways in ccRCC.

### 2.10 Statistical Analysis

R software (version 4.3.1) was utilized for all statistical analyses. For two‐group comparisons, Student's *t*‐test or Wilcoxon rank‐sum test was used as appropriate. For comparisons involving more than two groups, one‐way ANOVA or the Kruskal–Wallis test was used as appropriate. The chi‐square test or Fisher's exact test was applied to assess the connection between RPL28 expression and clinicopathological features. Kaplan–Meier curves and Cox regression models were implemented for survival analyses, whereas Pearson's or Spearman's correlation analysis was performed to investigate associations between variables. For immune‐infiltration and drug‐sensitivity analyses involving multiple comparisons, *p* values were adjusted using the Benjamini‐Hochberg method. Wound‐healing assays were quantified using ImageJ, and group comparisons were performed using Student's *t*‐test. Statistical significance was defined as a two‐sided *p* value < 0.05.

## 3 Results

### 3.1 RPL28 Expression in ccRCC

**FIGURE 1:**
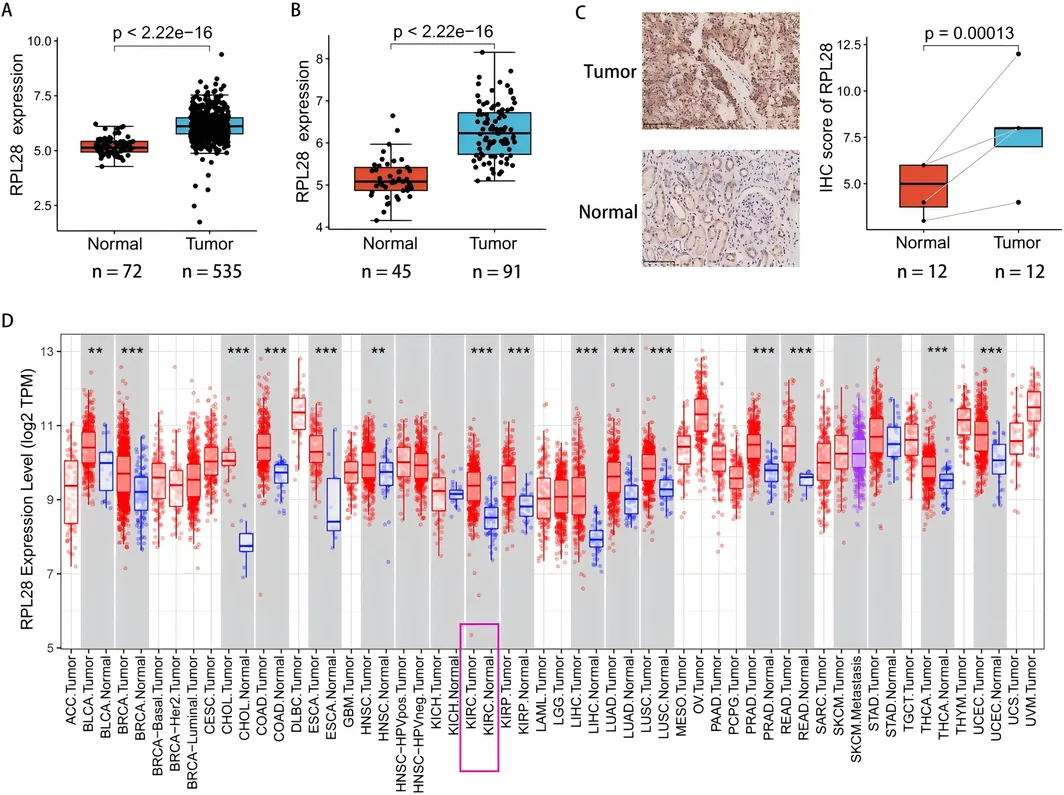
RPL28 expression is markedly upregulated in ccRCC. (A) Analysis of RPL28 expression within the TCGA ccRCC cohort demonstrates markedly elevated RPL28 levels in tumor tissues relative to normal kidney tissues. (B) Validation of RPL28 overexpression in ccRCC utilizing data derived from the International Cancer Genome Consortium (ICGC). (C) Immunohistochemical analysis confirms elevated RPL28 expression in real‐world ccRCC samples relative to adjacent normal tissues. (D) Pan‐cancer analysis of RPL28 expression employing the TIMER2.0 database. *p < 0.05, **p < 0.01, ***p < 0.001.

RPL28 expression in ccRCC was assessed employing data from The Cancer Genome Atlas (TCGA) cohort, which revealed markedly elevated RPL28 levels in ccRCC tissues relative to normal kidney tissues (*p* < 0.001) (Figure 1A). This finding was further validated using an independent dataset from the ICGC RECA‐EU cohort, which also showed a similar increase in RPL28 expression (*p* < 0.001) (Figure 1B). IHC examination of 12 paired ccRCC and adjacent normal tissue specimens validated the elevated expression of RPL28 at the protein level (*p* < 0.001) (Figure 1C). Furthermore, a pan‐cancer analysis conducted via the TIMER2.0 database identified RPL28 overexpression in multiple other cancers, including breast, bladder, cholangiocarcinoma, colon adenocarcinoma, liver hepatocellular carcinoma, and lung adenocarcinoma (Figure 1D).

### 3.2 Link Between RPL28 Expression and Clinicopathological Characteristics

**FIGURE 2:**
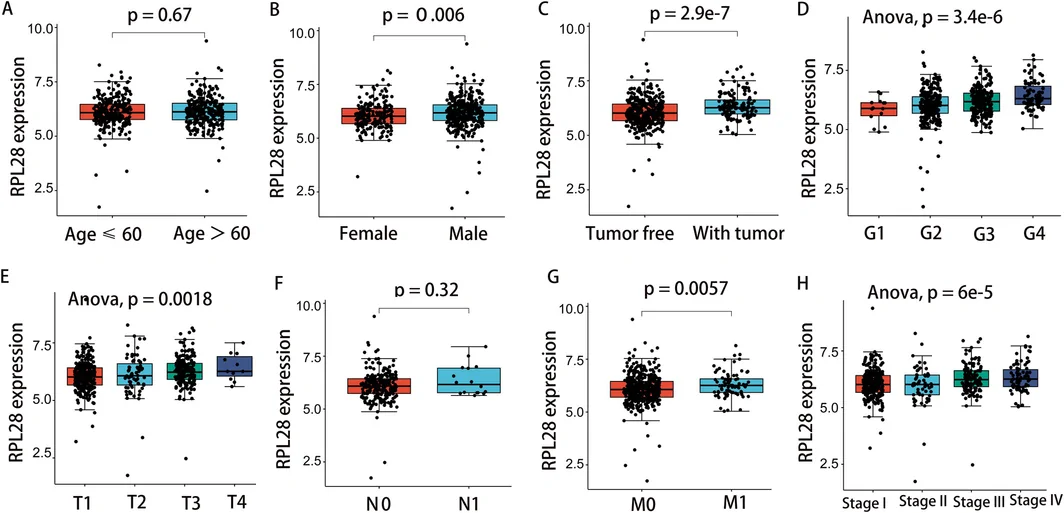
The link between RPL28 expression and clinicopathological characteristics of patients with ccRCC. (A–H) Comparison of RPL28 expression levels based on patient age, gender, tumor status, histological grade, *T* stage, *N* stage, *M* stage, and TNM stage. Two‐group comparisons (A–C, F, G) were performed using Student's *t*‐test or Wilcoxon rank‐sum test as appropriate. Multi‐group comparisons (D, E, H) were performed using one‐way ANOVA or Kruskal–Wallis test as appropriate.

To investigate the connection between RPL28 expression and clinicopathological features in ccRCC, the TCGA cohort comprising 535 ccRCC patients was split into high‐ and low‐RPL28 expression groups per the median expression value (Table 1). Enhanced RPL28 expression demonstrated significant correlations with male gender (*p* = 0.006), tumor presence (*p* < 0.001), elevated histological grade (*p* < 0.001), advanced *T* stage (*p* = 0.0018), distant metastases (*p* = 0.0057), and advanced TNM stage (*p* < 0.001). No marked connection was found between RPL28 expression and patient age (*p* = 0.67) or *N* stage (*p* = 0.32) (Figure 2A–H).

**TABLE 1:** Correlations between the expression of RPL28 and clinicopathologic characteristics in ccRCC.

Characteristic	*n* (%)	Expression of RPL28 (%)		*p*
		High	Low	
Total	535 (100)	267 (49.91)	268 (50.09)	
Age				0.604
≤ 60 years	267 (49.91)	130 (48.7)	137 (51.1)	
> 60 years	268 (50.09)	137 (51.3)	131 (48.9)	
Gender				0.014
Female	186 (34.77)	79 (29.6)	107 (39.9)	
Male	349 (65.23)	188 (70.4)	161 (60.1)	
Cancer status				< 0.001
Tumor free	336 (62.8)	142 (53.2)	194 (72.4)	
With tumor	148 (27.66)	99 (37.1)	49 (18.3)	
Unknown	51 (9.53)	26 (9.7)	25 (9.3)	
Grade				0.001
G1	14 (2.62)	6 (2.2)	8 (3.0)	
G2	231 (43.18)	99 (37.1)	132 (49.3)	
G3	207 (38.69)	110 (41.2)	97 (36.2)	
G4	75 (14.02)	51 (19.1)	24 (9.0)	
Unknown	8 (1.5)	1 (0.4)	7 (2.6)	
*T* stage				0.021
T1	275 (51.4)	123 (46.1)	152 (56.7)	
T2	70 (13.08)	33 (12.4)	37 (13.8)	
T3	179 (33.46)	103 (38.6)	76 (28.4)	
T4	11 (2.06)	8 (3.0)	3 (1.1)	
*N* stage				1
N0	240 (44.86)	118 (44.2)	122 (45.5)	
N1	16 (2.99)	8 (3.0)	8 (3.0)	
Unknown	279 (52.15)	141 (52.8)	138 (51.5)	
*M* stage				0.026
M0	424 (79.25)	200 (74.9)	224 (83.6)	
M1	78 (14.58)	48 (18.0)	30 (11.2)	
Unknown	33 (6.17)	19 (7.1)	14 (5.2)	
Stage				0.007
I	269 (50.28)	120 (44.9)	149 (55.6)	
II	58 (10.84)	24 (9.0)	34 (12.7)	
III	123 (22.99)	70 (26.2)	53 (19.8)	
IV	82 (15.33)	51 (19.1)	31 (11.6)	
Unknown	3 (0.56)	2 (0.7)	1 (0.4)	

Abbreviation: ccRCC, clear cell renal cell carcinoma.

### 3.3 The Prognostic Significance of RPL28 in ccRCC

**FIGURE 3:**
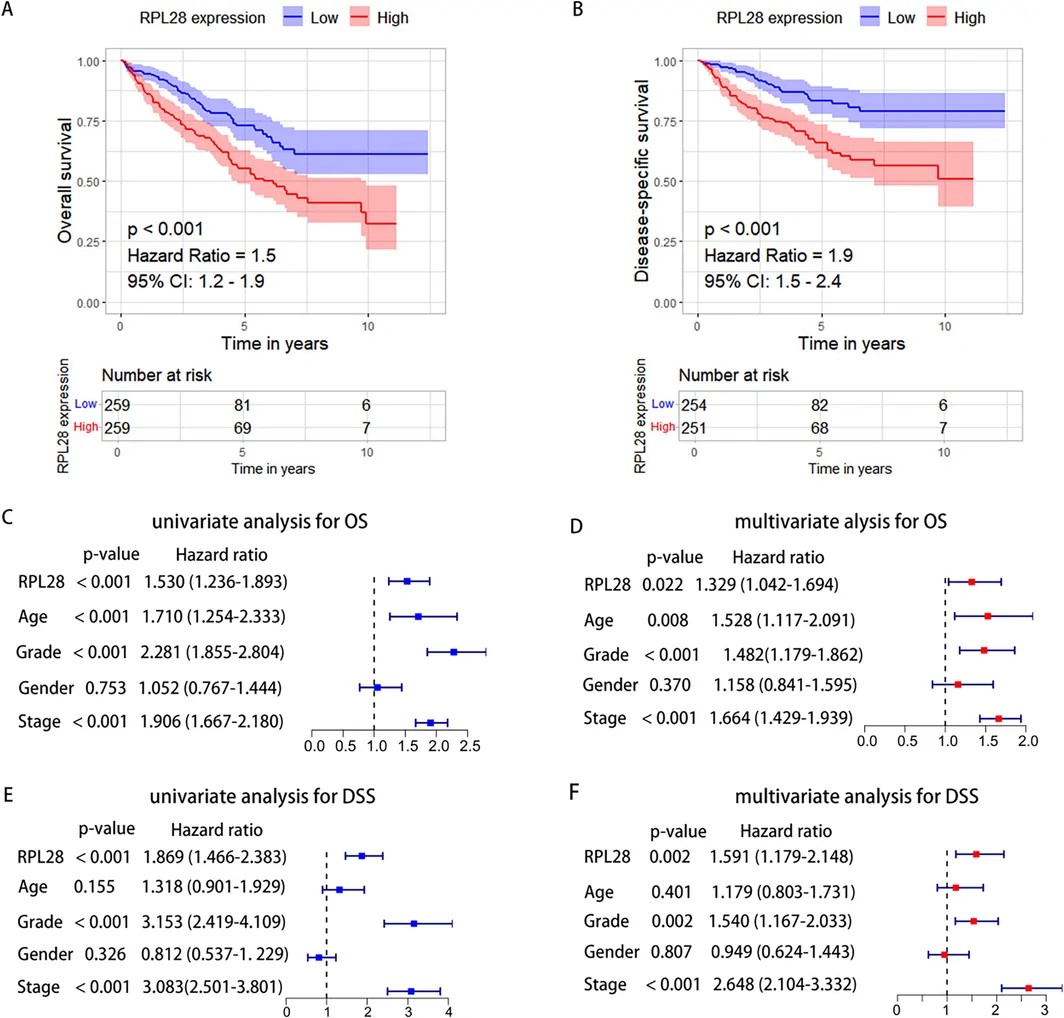
Prognostic value of RPL28 expression in ccRCC. (A, B) Kaplan–Meier survival curves show that higher RPL28 expression is linked to poorer OS and DSS in patients with ccRCC. (C, D) Univariate and multivariate Cox regression analyses indicate that RPL28 expression is an independent prognostic factor for OS. (E, F) Univariate and multivariate Cox regression analyses demonstrate that RPL28 expression is an independent predictor for DSS. ccRCC, clear cell renal cell carcinoma; DSS, disease‐specific survival; OS, overall survival.

Kaplan–Meier survival analysis was executed to evaluate the link between RPL28 expression and patient outcomes in ccRCC. Elevated RPL28 levels demonstrated significant correlation with inferior overall survival (OS) (Hazard Ratio [HR] = 1.5, *p* < 0.001) and disease‐specific survival (DSS) (HR = 1.9, *p* < 0.001) (Figure 3A,B). Univariate and multivariate Cox regression analyses were executed to ascertain independent prognostic factors. Univariate analysis revealed significant associations between RPL28 expression, patient age, histological grade, and TNM stage with OS in patients with ccRCC (Figure 3C). Multivariate analysis confirmed these findings (Figure 3D). However, only RPL28 expression, histological grade, and TNM stage were independently associated with DSS in both univariate and multivariate models (Figure 3E,F). These results highlight RPL28 expression, along with histological grade and TNM stage, as independent prognostic indicators for both OS and DSS, whereas age was recognized as an independent factor exclusively for OS in ccRCC.

**FIGURE 4:**
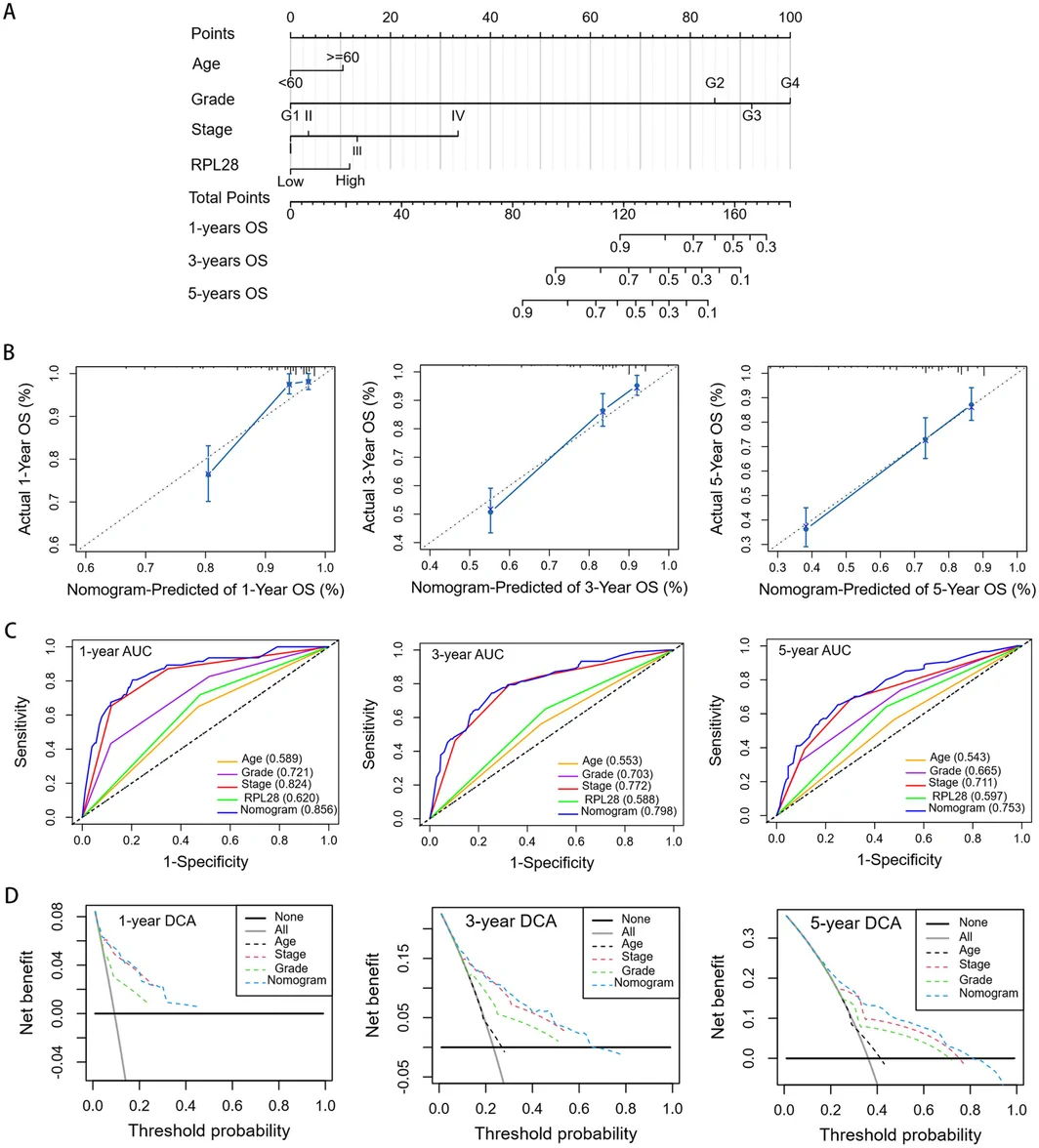
Nomogram utilizing RPL28 expression for overall survival (OS) prediction in patients with ccRCC. (A) A nomogram model was developed to predict the 1‐, 3‐, and 5‐year OS of patients with ccRCC utilizing RPL28 expression. (B) Calibration plots generated using 500 bootstrap resampling iterations demonstrate that the nomogram predictions closely correspond with the observed OS rates. (C) Time‐dependent ROC analysis shows that the nomogram outperforms individual prognostic factors in predicting 1‐, 3‐, and 5‐year OS. (D) Decision curve analysis indicates that the nomogram provides greater net benefits than individual prognostic factors.

Based on Cox regression analysis outcomes, nomograms were developed to forecast the 1‐, 3‐, and 5‐year OS probabilities for ccRCC patients (Figure 4A). Calibration plots revealed excellent concordance between nomogram‐predicted survival probabilities and ideal predictions (Figure 4B). Time‐dependent ROC analysis demonstrated that the area under the curve (AUC) for 1‐, 3‐, and 5‐year OS predictions reached 0.856, 0.798, and 0.753, respectively, surpassing individual independent factors (Figure 4C). Additionally, decision curve analysis (DCA) revealed that the nomogram offered superior net benefit for ccRCC patients relative to predictions using individual factors (Figure 4D). These observations indicate that RPL28 could function as a valuable prognostic biomarker for ccRCC.

### 3.4 Association Between RPL28 Expression and ccRCC Driver‐Gene Mutation Status

**FIGURE 5:**
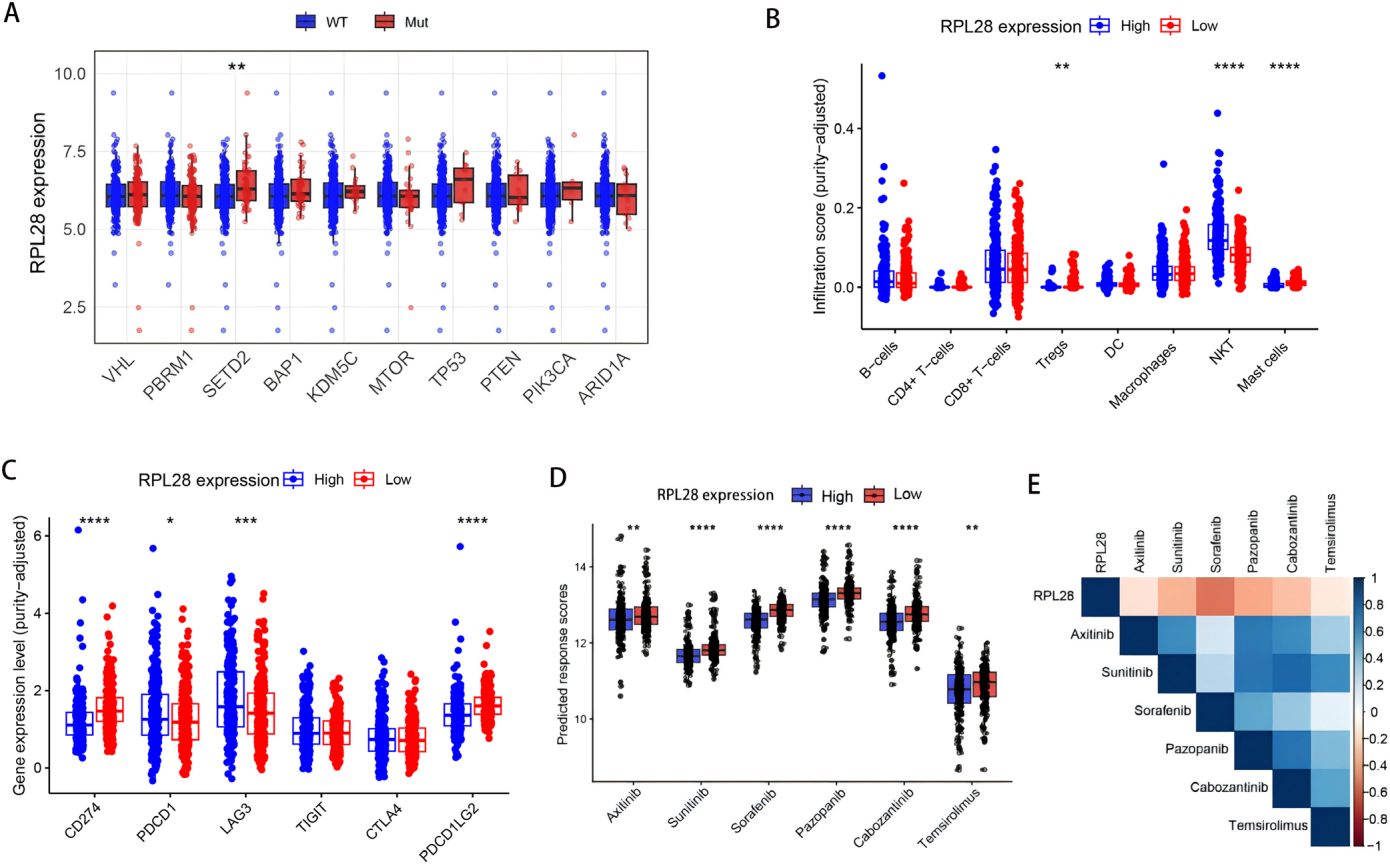
Driver‐gene mutation analysis, tumor‐purity‐adjusted immune infiltration, and drug‐sensitivity prediction according to RPL28 expression in ccRCC. (A) Association between RPL28 expression and the mutation status of representative ccRCC driver genes. (B) Differences in tumor‐purity‐adjusted immune‐cell infiltration scores between the RPL28‐high and RPL28‐low groups, as estimated by xCell. (C) Differences in tumor‐purity‐adjusted immune‐checkpoint‐related gene‐expression levels between the RPL28‐high and RPL28‐low groups. (D) Differences in predicted response scores of selected targeted agents between the RPL28‐high and RPL28‐low groups. Lower predicted response scores indicate greater predicted drug sensitivity. (E) Heatmap showing Spearman correlations between RPL28 expression and predicted response scores of selected targeted agents. Negative correlations indicate that higher RPL28 expression is associated with greater predicted sensitivity. *p < 0.05, **p < 0.01, ***p < 0.001, and ****p < 0.0001.

To investigate whether RPL28 expression was associated with major molecular subtypes of ccRCC, we compared RPL28 expression between mutant and wild‐type tumors for common ccRCC driver genes. Among the analyzed genes, RPL28 expression was significantly higher in SETD2‐mutant tumors than in SETD2‐wild‐type tumors (*p* < 0.01, Figure 5A). No significant difference in RPL28 expression was observed according to the mutation status of BAP1 or PBRM1. Similarly, RPL28 expression did not differ significantly between mutant and wild‐type groups for VHL, KDM5C, MTOR, TP53, PTEN, PIK3CA, or ARID1A. These results suggest that elevated RPL28 expression may be associated with a specific SETD2‐mutant molecular context in ccRCC, rather than being broadly explained by common 3p alterations such as BAP1 or PBRM1 mutations.

### 3.5 Association Between RPL28 Expression and Immune Infiltration in ccRCC

Because ccRCC is characterized by an immune‐active tumor microenvironment and is clinically responsive to immunotherapy, we investigated whether RPL28 expression was associated with immune infiltration after tumor‐purity adjustment. The purity‐adjusted xCell analysis showed significant differences in selected immune‐cell populations between the RPL28‐high and RPL28‐low groups, including Tregs (*p* < 0.01), NKT cells (*p* < 0.0001), and mast cells (*p* < 0.0001). In contrast, no significant differences were observed for B cells, CD4+ T cells, CD8+ T cells, dendritic cells, or macrophages after tumor‐purity adjustment (Figure 5B). Notably, MDSCs were not available as a directly estimated immune‐cell population in the xCell output used in this study; therefore, future studies using immune‐deconvolution tools or experimental approaches capable of evaluating MDSCs, such as multiplex immunohistochemistry, flow cytometry, or single‐cell RNA sequencing, will be necessary.

We further analyzed tumor‐purity‐adjusted expression levels of representative immune‐checkpoint‐related genes. The RPL28‐high group exhibited higher expression of PDCD1 (*p* < 0.05) and LAG3 (*p* < 0.001), whereas CD274 (*p* < 0.0001) and PDCD1LG2 (*p* < 0.0001) were expressed at lower levels. No significant differences were observed for TIGIT or CTLA4 after tumor‐purity adjustment (Figure 5C). These results suggest that RPL28 expression is associated with selected immune‐microenvironmental features in ccRCC, although these findings should be interpreted cautiously because they are based on computational analyses of bulk RNA‐seq data.

### 3.6 Association Between RPL28 Expression and Drug Sensitivity Prediction

To further explore the potential therapeutic relevance of RPL28 in ccRCC, we performed drug‐sensitivity prediction analysis based on the CTRP database. Patients were stratified into RPL28‐high and RPL28‐low groups according to the median RPL28 expression level. The selected agents included axitinib, sunitinib, sorafenib, pazopanib, cabozantinib, and temsirolimus. Lower predicted response values indicate greater predicted drug sensitivity. As shown in Figure 5D, the RPL28‐high group exhibited significantly lower predicted response values for several selected targeted agents than the RPL28‐low group, suggesting that tumors with high RPL28 expression may be more sensitive to these agents. In addition, correlation analysis showed that RPL28 expression was significantly associated with the predicted response values of selected agents (Figure 5E). These results indicate that RPL28 expression may have potential value for predicting targeted‐therapy sensitivity in ccRCC.

### 3.7 RPL28 Knockdown Suppresses the Malignant Behavior of ccRCC Cells In Vitro

**FIGURE 6:**
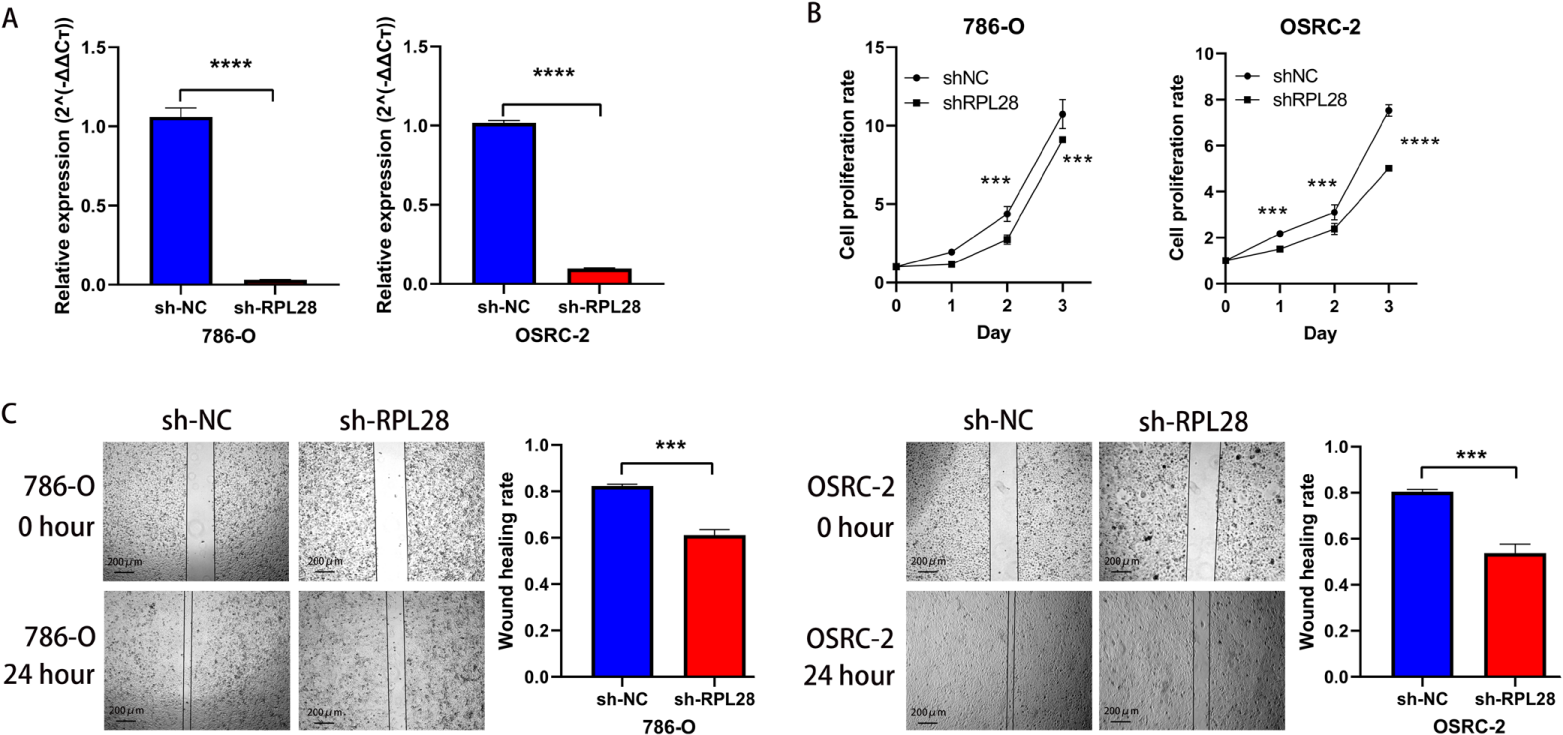
Knockdown of RPL28 inhibits the malignant phenotypes of ccRCC cells. (A) Validation of RPL28 silencing efficiency via qRT‐PCR. (B) CCK‐8 assay results show that RPL28 knockdown markedly diminishes the proliferation of 786‐O and OSRC‐2 cells. (C) Wound healing assays reveal that RPL28 knockdown markedly impairs the migration of 786‐O and OSRC‐2 cells. Scale bars in all wound‐healing images represent 200 μm. *p < 0.05, **p < 0.01, ***p < 0.001, and ****p < 0.0001.

To investigate the functional importance of RPL28 in ccRCC progression, stable RPL28 knockdown models were established in the 786‐O and OSRC‐2 cell lines via lentiviral shRNA‐mediated transfection. qPCR confirmed a marked reduction in RPL28 expression in the shRPL28 group versus the shNC group (Figure 6A). The CCK‐8 assay revealed that RPL28 silencing significantly impaired the proliferation of both 786‐O and OSRC‐2 cells (Figure 6B). Furthermore, the wound healing assay showed a notable decrease in the migratory capacity of 786‐O and OSRC‐2 cells following RPL28 knockdown (Figure 6C). These observations highlight the essential function of RPL28 in enhancing the malignant characteristics of ccRCC cells.

### 3.8 Functional Enrichment Analysis

**FIGURE 7:**
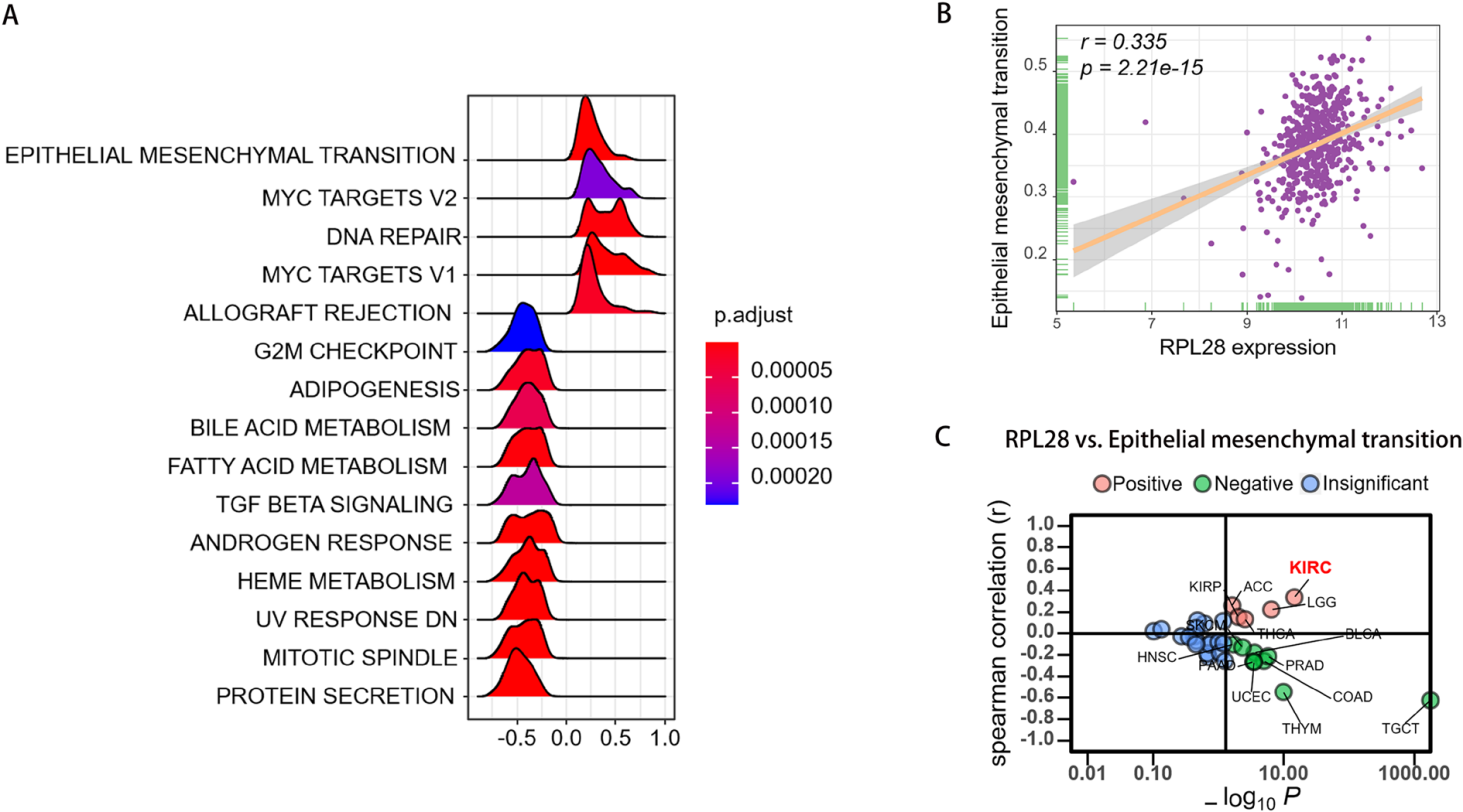
Functional enrichment analysis. (A) Gene set enrichment analysis reveals that elevated RPL28 expression links to multiple cancer‐associated pathways, encompassing epithelial‐mesenchymal transition (EMT), TGF‐β signaling, and MYC signaling. (B) Correlation analysis demonstrates a strong positive relationship between RPL28 expression and EMT in ccRCC. (C) Pan‐cancer analysis reveals a similar correlation between RPL28 expression and EMT across various other cancer types.

GSEA represents a well‐established methodology for functional enrichment analysis, detecting marked variations between two biological conditions within a specified gene set [24]. In this investigation, GSEA demonstrated that increased RPL28 expression exhibited strong associations with multiple cancer‐related pathways, encompassing epithelial–mesenchymal transition (EMT), the TGF‐β signaling pathway, and MYC signaling (Figure 7A). Detailed GSEA results, including NES, nominal *p* values, adjusted *p* values, and FDR q values, are provided in Table S1. EMT, a hallmark of tumor metastasis, is closely linked to both the TGF‐β and MYC pathways [25, 26, 27]. Subsequent analysis disclosed a marked positive connection between RPL28 expression and EMT (*r* = 0.335, *p* < 0.001) (Figure 7B). Notably, pan‐cancer analysis also demonstrated a robust association between RPL28 expression and EMT across multiple cancer types (Figure 7C).

## 4 Discussion

RCC remains the most prevalent and aggressive form of primary kidney cancer globally. ccRCC, the predominant subtype, accounts for approximately 75% of RCC cases and is characterized by high metastatic potential, poor prognosis, and resistance to conventional therapies such as chemotherapy and radiotherapy. Genetic alterations serve a pivotal function in ccRCC initiation and progression, highlighting the need to identify novel molecular pathways and prognostic biomarkers to improve treatment strategies and patient outcomes. This investigation performed a comprehensive analysis of high‐throughput RNA sequencing data from a ccRCC cohort in TCGA, with additional validation through IHC on clinical patient samples. Furthermore, in vitro loss‐of‐function assays were conducted to evaluate the biological role of RPL28. The results demonstrated that RPL28 expression was markedly linked to multiple clinicopathological parameters, OS, DSS, and key biological processes, encompassing cell proliferation and motility, in ccRCC. These observations indicate that RPL28 could function as a valuable prognostic biomarker and viable therapeutic target for ccRCC.

RPL28, a ribosomal protein from the L28E family, has been implicated in cancer progression. Prior research has demonstrated that RPL28 exhibits elevated expression across multiple cancer types, encompassing colorectal, esophageal, and liver cancers [9, 10, 28, 29]. Consistent with these findings, our analysis revealed markedly elevated RPL28 expression in patients with ccRCC. Furthermore, recent investigations have emphasized the prognostic significance of RPL28 across multiple cancer types. For instance, increased RPL28 levels have been linked to poorer survival outcomes in metastatic colorectal cancer, and its upregulation has been associated with reduced OS and unfavorable responses to azacitidine in patients with leukemia [8, 30]. This study confirmed that elevated RPL28 expression is strongly correlated with advanced histological grade, *T* stage, distant metastasis, and advanced TNM stage in ccRCC. Patients with elevated RPL28 expression demonstrated markedly worse OS and DSS.

Recent single‐cell sequencing, spatial transcriptomic, and multi‐omics studies have emphasized that ccRCC progression is shaped by substantial tumor‐cell heterogeneity, metabolic remodeling, immune‐microenvironmental organization, and molecular‐subtype diversity [17, 18, 31]. In addition to VHL‐driven pseudohypoxia and recurrent 3p tumor‐suppressor alterations, mutations in chromatin‐modifying genes such as PBRM1, SETD2, and BAP1 contribute to distinct biological and clinical phenotypes in ccRCC [11, 12]. In the present study, our exploratory mutation‐stratified analysis showed that RPL28 expression was significantly elevated in SETD2‐mutant tumors, whereas no significant association was observed for BAP1 or PBRM1 mutation status. SETD2 is involved in chromatin regulation and transcriptional control, and its alteration may be linked to broader changes in RNA processing, translational demand, and tumor‐cell state. However, our analysis does not prove that SETD2 directly regulates RPL28. Therefore, this result should be interpreted as hypothesis‐generating and more validated studies are needed in the future.

The immune‐infiltration analysis further extends the clinical relevance of RPL28 in ccRCC. ccRCC is among the most immune‐infiltrated solid tumors, yet the presence of immune cells does not necessarily indicate effective antitumor immunity. Recent single‐cell and spatial studies have shown that advanced ccRCC may contain exhausted or pro‐tumor immune states, spatially organized stromal niches, and tumor‐cell plasticity associated with immunotherapy response or resistance [13, 32, 33]. In this context, the tumor‐purity‐adjusted association between RPL28 expression and selected immune‐microenvironmental features suggests that RPL28 may participate in, or at least reflect, a tumor state connected with selective immune remodeling. Nevertheless, these findings are based on in silico deconvolution and should be validated in future studies using multiplex immunofluorescence, flow cytometry, or single‐cell/spatial transcriptomic datasets.

The pharmacogenomic analysis provides an additional translational perspective. VEGFR tyrosine‐kinase inhibitors remain important therapeutic agents in RCC, either historically as first‐line standards or currently as components of sequential treatment strategies [34]. Our exploratory drug‐sensitivity prediction suggests that RPL28 expression may be associated with differential sensitivity to several targeted agents. Nevertheless, predicted response values derived from CTRP‐trained models should be regarded as preliminary [35]. Future studies should examine whether RPL28 knockdown alters sensitivity to the relevant drugs in vitro and whether RPL28 expression predicts treatment outcomes in well‐annotated clinical cohorts.

As the most common RCC subtype, ccRCC originates from proximal tubular epithelium, with EMT playing a critical role in its metastatic progression [36]. Numerous studies have highlighted the impact of ribosomal proteins on EMT and tumor migration regulation. For instance, Liu et al. demonstrated elevated RPL23 levels in cisplatin‐resistant ovarian cancer, suggesting that its increased expression promotes chemotherapy resistance through EMT activation [37]. Similarly, Wen et al. [38] observed that RPS7 overexpression in prostate cancer enhances tumor growth and migration, while its suppression inhibits these processes by disrupting EMT. Zhang et al. [39] reported that high RPL14 expression in nasopharyngeal carcinoma promotes proliferation, migration, invasion, and EMT in cancer cells. In line with these findings, our GSEA analysis identified a strong correlation between RPL28 overexpression and EMT in ccRCC. Furthermore, wound healing assays confirmed that RPL28 knockdown significantly impaired ccRCC cell migration in vitro. Additionally, several cancer‐related pathways, including TGF‐β and MYC signaling, were markedly enriched in our GSEA results. Both TGF‐β and MYC are well‐established drivers of cancer initiation and progression, and they are known to regulate EMT and interact with ribosomal proteins [26, 27, 40, 41, 42]. Our findings suggest that RPL28 upregulation may act as a tumor‐promoting factor, likely contributing to the EMT process in ccRCC.

Despite the valuable insights offered by this research concerning the prognostic importance and biological function of RPL28 in ccRCC, several limitations require acknowledgment. Firstly, survival analysis primarily relied on TCGA cohort data, highlighting the need for multicenter studies to validate these findings. Secondly, these in silico findings should be interpreted cautiously because immune infiltration was inferred from bulk RNA‐seq deconvolution, MDSCs were not directly estimated in the present xCell output, and predicted drug sensitivity was not validated by drug‐treatment assays. Thirdly, the biological effects of RPL28 were assessed only through in vitro experiments, necessitating further in vivo studies for confirmation. Lastly, the precise molecular mechanisms by which RPL28 influences ccRCC progression remain unclear and warrant additional investigation.

## 5 Conclusion

In conclusion, RPL28 is overexpressed in ccRCC and is associated with unfavorable survival outcomes and malignant cellular phenotypes. RPL28 expression is also linked to EMT‐related signaling, selected immune‐microenvironmental features, and predicted therapeutic‐response phenotypes. These findings suggest that RPL28 may serve as a prognostic biomarker and potential therapeutic target in ccRCC, although further mechanistic and clinical validation is required.

## Author Contributions

**Runbiao Luo:** methodology, writing – original draft, formal analysis. **Guozhong Wu:** methodology, data curation, formal analysis. **Zhouzhou Xie:** methodology, data curation, formal analysis. **Yuchen Xiao:** methodology, data curation, formal analysis. **Wenbiao Zhu:** methodology, data curation, formal analysis. **Huiming Jiang:** conceptualization, writing – review and editing, project administration, funding acquisition, methodology.

## Funding

This investigation was supported by the Peiyu Project of the Meizhou People's Hospital (grant no. PY‐C2024035) and the Medical Science and Technology Research Foundation of Guangdong Province (grant no. B2025424).

## Ethics Statement

This study was approved by the Ethics Committee of Meizhou People's Hospital (approval number 2023‐C‐26) and followed the Declaration of Helsinki. Written informed consent was obtained from all participants.

## Consent

The authors have nothing to report.

## Conflicts of Interest

The authors declare no conflicts of interest.

## Supporting information


**Table S1:** GSEA results for all pathways displayed in Figure 7A.

## Data Availability

All data are available from the corresponding author on reasonable request.
